# Ceftaroline fosamil treatment patterns and outcomes in adults with community-acquired pneumonia: a real-world multinational, retrospective study

**DOI:** 10.1093/jacamr/dlae078

**Published:** 2024-05-27

**Authors:** Alex Soriano, Matteo Bassetti, Charalambos Gogos, Tristan Ferry, Raul de Pablo, Wajeeha Ansari, Michal Kantecki, Bernd Schweikert, Gustavo Luna, Francesco Blasi

**Affiliations:** Infectious Diseases Department, Hospital Clínic de Barcelona, CIBERINF, CIBER in Infectious Diseases, Barcelona, Spain; Infectious Diseases, Clinica Malattie Infettive, Ospedale Policlinico IRCCS San Martino and University of Genoa, Genoa, Italy; Divison of Infectious Diseases, Department of Internal Medicine, University of Patras, Patras, Greece; Infectious Diseases Department, Croix-Rousse Hospital, Hospices Civils de Lyon, Lyon, France; Department of Intensive Care, Hospital Universitario Ramón y Cajal, IRYCIS, University of Alcalá, Madrid, Spain; Pfizer Biopharmaceuticals Group, Pfizer, New York, NY, USA; Global Medical Affaris, Pfizer, Paris, France; Health Economics and Epidemiology, ICON plc, Munich, Germany; Health Economics and Epidemiology, ICON plc, Stockholm, Sweden; Respiratory Unit and Cystic Fibrosis Center, Fondazione IRCCS Cà Granda Ospedale Maggiore Policlinico Milan, Italy; Department of Pathophysiology and Transplantation, Università degli Studi di Milan, Italy

## Abstract

**Background:**

This multicentre, observational, retrospective chart review study assessed ceftaroline fosamil treatment patterns and outcomes in adults hospitalized with community-acquired pneumonia (CAP) in usual care settings.

**Methods:**

Anonymized patient data were extracted from hospital records of adults with CAP who received ≥4 consecutive IV ceftaroline fosamil doses at sites in Brazil, Colombia, France, Greece, Italy, Russia and Spain.

**Results:**

The dataset included 185 patients (58.9% male; mean age 62.2 years), of whom 128 (69.2%) had severe CAP defined by CURB-65, PSI/PORT score or physician judgement. *Streptococcus pneumoniae* (*n *= 44; 23.8%) and *Staphylococcus aureus* [MSSA (*n *= 15) and MRSA (*n *= 14)] were the most frequently identified pathogens. Clinical response occurred in 151 (81.6%) patients overall, and in 104 (81.3%) severe CAP patients. Response within ≤4 and >4 days occurred in 79 (42.7%) and 62 (33.5%) patients (unknown, *n *= 10), respectively. Twenty (10.8%) patients required readmission within 30 days. Thirty-day all-cause mortality rates were 9.7% (*n *= 18) overall and 10.2% (*n *= 13) in severe CAP. In sensitivity analysis using ICU admission as a more objective marker of severe CAP (*n *= 75), clinical response and 30 day mortality occurred in 57 (76.0%) and 10 (13.3%) patients, respectively. Overall, clinical response to ceftaroline fosamil was associated with >60% shorter length of ICU stay (3.6 versus 9.2 days), and >30% lower hospital costs ($8449 versus $12 559) versus non-responders.

**Conclusions:**

Ceftaroline fosamil was effective in treating adults with CAP, including severe CAP, in Europe and Latin America. Clinical response to ceftaroline fosamil was associated with reductions in healthcare resource use compared with non-responders.

## Introduction

Community-acquired pneumonia (CAP) is associated with considerable morbidity, mortality and healthcare resource use.^1^ CAP is the most common cause of death due to infection in Europe;^2^ mortality is associated with advanced age, comorbid conditions and CAP severity.^3^ The emergence of antimicrobial resistance, particularly among *Streptococcus pneumoniae* isolates, has led to current empirical antimicrobial treatment options for CAP being compromised in some regions, including some parts of Latin America.^4,5^ There is therefore a need, particularly in patients at risk of treatment failure, for alternative therapies that improve empirical treatment success rates.

Treatment guidelines for CAP in Europe and Latin America recommend empirical treatment at the outset, with factors such as disease severity, individual risk of mortality, and susceptibility patterns of pathogens against available antibiotics in the geographical region contributing to decisions regarding treatment choice.^6,7^

Ceftaroline is a fifth-generation cephalosporin with *in vitro* activity against Gram-positive pathogens, including MSSA and MRSA, streptococci, including MDR *S. pneumoniae*, and common (non-ESBL-producing) Gram-negative organisms (excluding *Pseudomonas aeruginosa*).^8,9^

Ceftaroline fosamil, the prodrug of active ceftaroline, has been shown in randomized multinational trials and observational studies to be an effective treatment for patients hospitalized with CAP or complicated skin and soft tissue infections (cSSTIs).^10–17^ For patients with CAP, ceftaroline fosamil has demonstrated clinical superiority to ceftriaxone, a standard treatment in this setting, and simulation data indicate that at standard doses it achieves greater pharmacokinetic/pharmacodynamic target attainment than ceftriaxone or levofloxacin against common CAP pathogens.^18,19^

This study assessed treatment use patterns, healthcare resource use and treatment outcomes in adult patients hospitalized with CAP or cSSTI treated with ceftaroline fosamil in a usual care setting in Europe and Latin America. Results for patients with CAP are presented here.

## Material and methods

### Ethics

The study was approved by the relevant local independent ethics committees, and/or institutional review boards approved the final study protocol for each of the sites in this multicentre study (details for each site provided in the Supplementary Methods, available as Supplementary data at *JAC-AMR* Online). Informed consent was waived for the majority of sites due to the retrospective nature of the research; for the remaining sites, informed consent forms were obtained from patients (Supplementary Methods). The study was conducted according to the guidelines of the Declaration of Helsinki.

### Study design and patients

This was a multicentre, observational, retrospective chart review study (ClinicalTrials.gov identifier: NCT04198571) conducted in Spain, Greece, Russia, Italy, France, Colombia and Brazil. Hospital sites identified all patients dispensed ≥4 IV doses of ceftaroline fosamil on or before 31 May 2019 by querying their hospital pharmacy dispensing records. Records of potentially eligible patients were then screened manually by site staff to identify patients who met all inclusion criteria without meeting any of the exclusion criteria. Inclusion criteria were: adult patients (≥18 years old) with CAP who had received ≥4 consecutive IV doses of ceftaroline fosamil on or before 31 May 2019. Diagnostic criteria for CAP are included in the Supplementary Methods. Severity of CAP was determined according to pneumonia severity index (PSI)/pneumonia patient outcomes research team (PORT) risk classification, confusion, urea nitrogen, respiratory rate, blood pressure, and ≥65 years of age (CURB-65) score or other prognostic scoring system as entered by the physician on the patient case report form (CRF). A sensitivity analysis, using patients who were admitted to the ICU (duration >0 days) as an objective marker of severe CAP, was also conducted.

Patients were excluded if their medical records were missing documentation of CAP according to the diagnostic criteria, details of ceftaroline fosamil dosing, success/failure of treatment, reason for discontinuation of treatment, or discharge date and status. Patients who developed signs and symptoms of sepsis or septic shock (definitions provided in the Supplementary Methods) during the index hospitalization were included in the study, but patients who developed hospital-acquired pneumonia or ventilator-associated pneumonia ≥48 h after the index hospital admission were not included.

### Analysis

Relevant data, including patient, disease and treatment characteristics, and clinical and healthcare resource use outcomes data, were extracted from hospital records of eligible patients from 3 months before the index hospital admission until 30 days after hospital discharge date or death, whichever occurred first.

Treatment response was defined as demonstrating clinical stability (defined according to the IDSA guidelines^20^ as temperature of ≤37.8°C, heart rate of ≤100 beats/min, respiratory rate of ≤24 breaths/min, systolic blood pressure of ≥90 mmHg, oxygen saturation of ≥90%, and confusion/disorientation recorded as absent) and clinical improvement [defined as improvement of at least one of four symptoms present at baseline (i.e. cough, dyspnoea, pleuritic chest pain or sputum production) with worsening of none]. Clinical cure was defined as no further IV antibiotic, switch to an oral antibiotic, or IV antibiotic treatment streamlining/de-escalation at any time after the index dose, prior to hospital discharge. Clinical failure was defined as switch to another IV antibiotic due to an adverse reaction, drug–drug interaction, insufficient response, or a microbiological diagnosis indicating that the pathogen was not susceptible to ceftaroline fosamil.

Patient characteristics, clinical management and treatment responses were summarized descriptively; healthcare resource use was evaluated by treatment response to ceftaroline fosamil. No *a priori* hypotheses were specified; a formal sample size calculation was therefore not applicable.

## Results

### Patient and disease characteristics

A total of 185 patients with CAP were included [58.9% male; mean age 62.2 years (excluding three patients aged >90 years)], the majority at sites in Spain, Greece, Italy and Russia (Table 1). The most frequent comorbidities present at index hospitalization were diabetes mellitus (22.7%), COPD (17.3%) and congestive heart failure (17.3%).

**Table 1.** Demographic and baseline characteristics and isolated pathogens of patients with CAP at index hospitalization

Characteristic	Patients (*n* = 185)
Age, years, *n* (%)	
>90	3 (1.6)
≤90	182 (98.4)
Mean (SD)	62.2 (18.9)
≤65	90 (48.6)
>65	95 (51.4)
Sex, *n* (%)	
Male	109 (58.9)
Female	76 (41.1)
Country, *n* (%)	
France	16 (8.6)
Greece	53 (28.6)
Italy	24 (13.0)
Spain	54 (29.2)
Russia	25 (13.5)
Brazil	6 (3.2)
Colombia	7 (3.8)
Weight, kg, mean (SD)^a^	74.0 (19.4)
BMI, kg/m^2^, mean (SD)^b^	26.1 (6.2)
Type of residence/cohabitation pre-index admission, *n* (%)^c^	
Nursing home or extended-care facility	8 (4.3)
Living independently	122 (65.9)
Living with care support (family, friend, hired support)	46 (24.9)
Other	1 (0.5)
Smoking habits, *n* (%)	
Non-smoker	71 (38.4)
Ex-smoker (stopped ≥365 days ago)	38 (20.5)
Occasional smoker (<1 tobacco product per day)	4 (2.2)
Habitual smoker (≥1 tobacco products per day)	38 (20.5)
Unknown	34 (18.4)
qSOFA conducted, *n* (%)	
Yes	74 (40.0)
qSOFA component assessment, *n* (%)	
Glasgow coma scale <15	15 (20.3)
Systolic blood pressure <100 mmHg	15 (20.3)
High respiration rate (≥22 breaths per min)	45 (60.8)
Patient required isolation, *n* (%)	
Yes	20 (10.8)
Duration of isolation, days, mean (SD)	16.5 (14.0)
Isolated pathogens, *n* (%)	
*S. pneumoniae*	44 (23.8)
*S. aureus* (all)	33 (17.8)
MRSA	14 (7.6)
MSSA	15 (8.1)
Methicillin susceptibility not reported	4 (2.2)
*Klebsiella pneumoniae*	3 (1.6)
*Haemophilus influenzae*	3 (1.6)
*Legionella* spp.^d^	2 (1.1)
*Escherichia coli*	2 (1.1)
*Haemophilus parainfluenzae*	1 (0.5)
*P. aeruginosa*^d^	1 (0.5)
Other/unknown/none of the above	113 (61.1)

^a^ *n* = 90 (data not available for 95 patients).

^b^ *n* = 79 (data not available for 106 patients).

^c^ *n* = 177 (data not available for eight patients).

^d^Not susceptible to ceftaroline fosamil.

In total, 128 (69.2%) patients were graded as having severe CAP; severity assessment was unknown in 10 patients (5.4%). Where prognostic scoring system information was available, CAP severity was determined by PSI/PORT score in 18 patients (mean score: 90.1) and by CURB-65 score in 41 patients (mean score: 2.2). Severity of CAP was attributed to being determined by physician judgement for the remaining patients. In total, 75 (40.5%) patients had ICU admission ≥1 day and were included in the sensitivity analyses of patients with severe CAP.

Ten patients (5.4%) were admitted with a recurrent episode of CAP; 38 (20.5%) patients had sepsis, 16 (8.6%) had severe sepsis, 38 (20.5%) had septic shock and 56 (30.3%) required mechanical ventilation. Results of quick sepsis-related organ failure assessment (qSOFA) are shown in Table 1.

The most frequently identified pathogens were *S. pneumoniae* (23.8%), *Staphylococcus aureus*, including methicillin-resistant and -susceptible strains (17.8%), and ‘other microorganisms’ (14.1%; Table 1).

### Treatment characteristics

Data on ceftaroline fosamil treatment during the index hospitalization are shown in Table 2. Median (range) ceftaroline fosamil treatment duration was 7 (2–35) days at daily doses of 1200 (200–1800) mg.

**Table 2.** Details of ceftaroline fosamil treatment during index hospitalization

Treatment variable	Patients (*n* = 185)
Ceftaroline fosamil line of therapy, *n* (%)	
1	50 (27.0)
2	49 (26.5)
3	46 (24.9)
≥4	40 (21.5)
Duration of treatment, days, median (range)	7 (2–35)
Time from admission to first dose, days, median (range)	1.9 (0–36)
Time from symptom onset to first dose, days, median (range)	6 (0–38)
Daily dose, mg, median (range)	1200 (200–1800)
Treatment type, *n* (%)^a^	
Empirical	138 (74.6)
Definitive/specific	41 (22.0)
Monotherapy/combination therapy, *n* (%)	
Monotherapy	56 (30.3)
Combination therapy^b^	129 (69.7)
Aminoglycoside	3 (2.3)
β-lactam	8 (6.2)
Carbapenem	7 (5.4)
Ceftriaxone	5 (3.9)
Cephalosporin	1 (0.8)
Glycopeptide	5 (3.9)
Macrolide	2 (1.6)
β-lactam/combination	36 (27.9)
Sulphonamide	57 (44.2)
Clindamycin	5 (3.9)
Other	5 (3.9)
Administration location, *n* (%)	
ICU	66 (35.7)
General ward	115 (62.2)
At home	1 (0.5)
Medical clinic	30 (16.2)

^a^ *n* = 179 (data not available for six patients).

^b^ *n* = 99 (data not available for 30 patients).

Ceftaroline fosamil was used empirically (i.e. in the absence of definitive microbial pathogen identification) in 138 (74.6%) patients, and as first-line therapy in 50 (27.0%) patients. In total, 134 patients (72.4%) received another antibiotic treatment for the index infection prior to receiving ceftaroline fosamil; across all treatment lines the most frequently administered were quinolones and ceftriaxone (Table S1). Quinolones were the agents most frequently given as first-line therapy [*n* = 39/134 (29.1%)]. The median (range) number of lines of therapy of other antibiotics given prior to ceftaroline fosamil was 2 (1–8) (Table S1). Fifty-six (30.3%) patients received ceftaroline fosamil monotherapy. When used in combination, the most frequently coadministered antibiotics were sulphonamides (Table S2).

In total, 99 (53.5%) patients had their treatment modified following treatment with ceftaroline fosamil; where reasons for treatment switch were provided, the most frequently recorded were lack of efficacy [*n* = 39 (39.4%)] and results of susceptibility test/pathogen identification [*n* = 26 (26.3%)] (Table S3). The antibiotics most frequently administered after switching from ceftaroline fosamil were quinolones [*n* = 45 (45.5%)], clindamycin [*n* = 32 (32.3%)], and β-lactam/combination [*n* = 27 (27.3%)] (Table S3).

### Clinical outcomes

Clinical response occurred in 151 (81.6%) patients, among whom response within ≤4 days and >4 days occurred in 79 (52.3%) and 62 (41.1%), respectively (Table 3). Clinical response in those with severe CAP occurred in 104 of 128 (81.3%) patients based on physician judgement, and in 57 of 75 (76.0%) patients who were admitted to the ICU (Table 3).

**Table 3.** Clinical outcomes of ceftaroline fosamil treatment

Outcome measure	All patients (*n* = 185)	Patients with severe CAP (*n* = 128)	Patients admitted to the ICU (*n* = 75)
Treatment response, *n* (%)			
Clinical response^a^	151 (81.6)	104 (81.3)	57 (76.0)
Clinical failure	34 (18.4)	24 (18.8)	18 (24.0)
Reason for failure			
Insufficient response	21 (61.8)	16 (66.7)	10 (55.6)
Death due to index infection	7 (20.6)	6 (25.0)	4 (22.2)
Death due to other	2 (5.9)	1 (4.2)	1 (5.6)
Relapse or recurrence	2 (5.9)	0	1 (5.6)
Unknown	2 (5.9)	1 (4.2)	2 (11.1)
Time to clinical response, days, mean (SD)^b^	5.0 (3.5)	5.7 (3.7)	5.7 (3.9)
Early clinical response, *n* (%)			
>4 days	62/151 (33.5)	54/104 (51.9)	25/57 (43.9)
≤4 days	79/151 (42.7)	46/104 (44.2)	28/57 (49.1)
Unknown	10/151 (5.4)	4/104 (3.8)	4/57 (7.0)
Clinical cure achieved, *n* (%)^c,d^			
Yes	115 (62.2)	78 (60.9)	44 (48.7)
No	35 (18.9)	25 (19.5)	13 (17.3)
Time to clinical cure, days, mean (SD)^e^	7.8 (4.3)	8.1 (4.2)	7.2 (3.3)
Time to clinical stability, days, mean (SD)	3.8 (2.9)	4.4 (3.0)	4.5 (3.1)
Time to clinical improvement, days, mean (SD)	4.5 (3.3)	5.1 (3.5)	5.0 (3.7)
Discharge status, *n* (%)			
Died in hospital	18 (9.7)	13 (10.2)	10 (13.3)
Discharged to a nursing home or extended-care facility	28 (15.1)	21 (16.4)	15 (20.0)
Discharged to independent living (with or without support)	138 (74.6)	93 (72.7)	50 (66.7)
Other	1 (0.5)	1 (0.8)	0 (0)
Re-hospitalized within 30 days of initial discharge, *n* (%)			
Yes	20 (10.8)	12 (9.4)	11 (14.7)
No	129 (69.7)	89 (69.5)	46 (61.3)
Unknown	36 (19.5)	27 (21.1)	18 (24.0)
Number of re-hospitalizations for those re-hospitalized, median (range)	1 (1–3)	1 (1–1)	1 (1–1)
Vital status at end of follow-up, *n* (%)^f^			
Patient still alive	126 (68.1)	86 (67.2)	57 (76.0)
Patient deceased	13 (7.0)	6 (4.7)	12 (16.0)
If deceased, duration from discharge, days, mean (SD)	269 (300.6)	311 (397.5)	473 (603.9)

^a^Defined as demonstrating clinical stability (defined according to the IDSA guidelines^20^ as temperature of ≤37.8°C, heart rate of ≤100 beats/min, respiratory rate of ≤24 breaths/min, systolic blood pressure of ≥90 mmHg, oxygen saturation of ≥90%, and confusion/disorientation recorded as absent) and clinical improvement [defined as improvement of at least one of four symptoms present at baseline (i.e. cough, dyspnoea, pleuritic chest pain or sputum production) with worsening of none].

^b^All patients, *n* = 141 (data not available for 10 patients); patients with severe CAP, *n* = 100 (data not available for four patients); patients admitted to the ICU, *n* = 53 (data not available for four patients).

^c^All patients, *n* = 150 (data not available for 35 patients); patients with severe CAP, *n* = 103 (data not available for 25 patients); patients admitted to the ICU, *n* = 57 (data not available for 18 patients).

^d^Defined as no further IV antibiotic, switch to an oral antibiotic, or IV antibiotic treatment streamlining/de-escalation at any time after the index dose, prior to hospital discharge.

^e^All patients, *n* = 108 (data not available for seven patients); patients with severe CAP, *n* = 74 (data not available for four patients); patients admitted to the ICU, *n* = 40 (data not available for four patients).

^f^All patients, *n* = 139 (data not available for 46 patients); ICU patients, *n* = 69 (data not available for six patients).

Clinical failure occurred in 34 (18.4%) patients; the most common reason for clinical failure was insufficient response (Table 3). Where known, the most common pathogens isolated at baseline for patients with clinical failure were *S. pneumoniae* [*n* = 6 (17.6%)], MRSA [*n* = 5 (14.7%)] and MSSA [*n* = 4 (11.8%)]. In total, 23 of 34 patients with clinical failure received antibiotic treatment for the index infection following ceftaroline fosamil; the most common agent received was clindamycin [*n* = 17 (50.0%)].

Clinical response was numerically higher, and with a numerically higher occurrence of early time to response, in patients receiving ceftaroline fosamil as first-line therapy compared with later lines of therapy (Table 4).

**Table 4.** Clinical outcomes by ceftaroline fosamil line of therapy

Outcome measure	All patients (*n* = 185)		Patients with severe CAP (*n* = 128)	
	First-line ceftaroline fosamil (*n* = 50)	Later-line ceftaroline fosamil (*n* = 135)	First-line ceftaroline fosamil (*n* = 35)	Later-line ceftaroline fosamil (*n* = 93)
Treatment response, *n* (%)				
Clinical response^a^	44 (88.0)	107 (79.3)	29 (82.9)	75 (80.7)
Clinical failure	6 (12.0)	28 (20.7)	6 (17.1)	18 (19.4)
Time to clinical response, days, mean (SD)	4.9 (3.3)	5.1 (3.6)	5.5 (3.4)	5.8 (3.8)
Early clinical response, *n* (%)				
>4 days	18/44 (40.9)	44/107 (41.1)	16/29 (55.2)	38/75 (50.7)
≤4 days	25/44 (56.8)	54/107 (50.5)	13/29 (44.8)	33/75 (44.0)
Unknown	1/44 (2.3)	9/107 (8.4)	0	4/75 (5.3)
Clinical cure achieved, *n* (%)				
Yes	33 (66.0)	82 (60.7)	22 (62.9)	56 (60.2)
No	10 (20.0)	25 (18.5)	6 (17.1)	19 (20.4)
Unknown	7 (14.0)	28 (20.7)	7 (20.0)	18 (19.4)
Time to clinical cure, days, mean (SD)	6.7 (4.0)	8.3 (4.4)	7.1 (4.5)	8.5 (4.0)
Time to clinical stability, days, mean (SD)	3.6 (2.4)	3.9 (3.1)	4.1 (2.4)	4.6 (3.2)
Time to clinical improvement, days, mean (SD)	4.9 (3.4)	4.4 (3.3)	5.4 (3.5)	4.9 (3.6)

^a^Defined as demonstrating clinical stability (defined according to the IDSA guidelines^20^ as temperature of ≤37.8°C, heart rate of ≤100 beats/min, respiratory rate of ≤24 breaths/min, systolic blood pressure of ≥90 mmHg, oxygen saturation of ≥90%, and confusion/disorientation recorded as absent) and clinical improvement [defined as improvement of at least one of four symptoms present at baseline (i.e. cough, dyspnoea, pleuritic chest pain or sputum production) with worsening of none].

Death due to the index infection occurred in seven (3.8%) patients. Thirty-day all-cause mortality occurred in 18 (9.7%) overall and in 13 (10.2%) patients with severe CAP based on physician judgement. In patients who were admitted to the ICU, 30 day mortality occurred in 10 (13.3%).

Of pathogens identified in ≥5 patients with microbial data available, those most frequently associated with mortality were MRSA, *S. pneumoniae* and MSSA (Table S4).

Survival rate was numerically higher in patients receiving ceftaroline fosamil as first-line therapy, compared with a later line of therapy (Table 5).

**Table 5.** Survival status by ceftaroline fosamil line of therapy

Ceftaroline fosamil line of therapy, *n*/*N* (%)	All patients^a^		Patients with severe CAP^b^	
	Deceased (*n* = 31)	Alive (*n* = 126)	Deceased (*n* = 19)	Alive (*n* = 86)
Line 1	1/41 (2.4)	40/41 (97.6)	1/27 (3.7)	26/27 (96.3)
Line 2	11/35 (31.4)	24/35 (68.6)	10/28 (35.7)	18/28 (64.3)
Line 3	12/42 (28.6)	30/42 (71.4)	4/22 (18.2)	18/22 (81.8)
Line 4+	7/39 (18.0)	32/39 (82.1)	4/28 (14.3)	24/28 (85.7)

^a^Data available for 157/185 patients.

^b^Data available for 105/128 patients.

Overall, 20 (10.8%) patients were readmitted to hospital within 30 days of initial discharge. Of those readmitted, the cause of readmission was the index infection in seven (35.0%) patients, and other reasons in 13 (65.0%) patients.

### Healthcare resource use

Overall mean (SD) duration of index hospitalization was 19.4 (18.3) days. Mean (SD) duration of ICU stay was 4.6 (8.5) days (Table 6). Clinical response to ceftaroline fosamil was associated with shorter length of stay in hospital (mean 18.3 versus 24.1 days) and in the ICU (mean 3.6 versus 9.2 days), as well as with lower hospital costs (>30%), compared with non-responders (Table 6). Breakdown of country-specific healthcare costs is shown in Table S5.

**Table 6.** Healthcare resource outcomes of ceftaroline fosamil treatment

Outcome measure	Clinical response to ceftaroline fosamil^a^	
	Response (*n* = 151)	No response (*n* = 34)
Length of stay, days, mean (SD)		
Hospital	18.3 (17.5)	24.1 (20.9)
ICU	3.6 (6.7)	9.2 (13.2)
Hospital costs, USD, mean (SD)		
Standard hospital^b^	8449.3 (12 581.6)	12 559.1 (13 908.4)
Advanced-level hospital^c^	23 031.7 (29 917.1)	35 961.6 (37 359.3)

USD, US dollars.

^a^Clinical response defined as demonstrating clinical stability (defined according to the IDSA guidelines^20^ as temperature of ≤37.8°C, heart rate of ≤100 beats/min, respiratory rate of ≤24 breaths/min, systolic blood pressure of ≥90 mmHg, oxygen saturation of ≥90%, and confusion/disorientation recorded as absent) and clinical improvement [defined as improvement of at least one of four symptoms present at baseline (i.e. cough, dyspnoea, pleuritic chest pain or sputum production) with worsening of none].

^b^Standard hospital cost: total time in hospital multiplied by per diem rate of standard hospital general ward.

^c^Advanced hospital cost: total time in hospital multiplied by per diem rate of hospitals providing the highest level of medical services.

## Discussion

This study assessed ceftaroline fosamil real-world treatment patterns and clinical outcomes in hospitalized adults with CAP in Europe and Latin America in a usual care setting. Ceftaroline fosamil provided effective treatment for patients with CAP, with numerically higher clinical response and survival rates in patients receiving ceftaroline fosamil as first-line therapy, compared with later lines of therapy.

In line with the protocol for this retrospective chart review study, severity of CAP was determined according to PSI/PORT risk classification, CURB-65 score, other prognostic scoring system, or ‘unknown’ as entered by the physician on the patient CRF. The ‘other’ category may have included the IDSA/American Thoracic Society (ATS) criteria,^21^ amongst others. However, of the 128 patients classified by the physician as having severe CAP, prognostic scoring system information was only available for a limited number; accordingly, a working definition based on CURB-65, PSI/PORT and physician judgement was used to define severe CAP. As the available data were insufficient to objectively classify CAP severity, a sensitivity analysis was conducted, using patients who were admitted to the ICU for any amount of time as an objective marker of severe CAP. In total, 75 (41%) patients had ICU admission and were included in the sensitivity analyses. The use of ICU admission, while not wholly objective due to institutional variations in admission practices and criteria, is endorsed by the European Respiratory Society (ERS), European Society of Intensive Care Medicine (ESICM), ESCMID and Latin American Thoracic Association (ALAT) as a surrogate indicator of severe CAP.^22^ Importantly, treatment response to ceftaroline fosamil was demonstrated regardless of CAP severity, occurring in 82% of patients overall, and in 81% of those with severe CAP based on physician judgement and 76% of patients who were admitted to the ICU.

Ceftaroline fosamil was given empirically in 75% of hospitalized patients with CAP, potentially reflecting physician suspicion of MRSA in these patients. In clinical practice, as the identity of aetiological CAP pathogens is often unknown, patients are typically diagnosed based on clinical signs and symptoms.^20,23^ Empirical therapy needs to be active against the most likely causative pathogens while not providing excessively broad antimicrobial coverage. As initial treatment failure is associated with longer hospital stays, higher mortality rates and increased healthcare costs,^24^ the appropriate choice of initial antibiotic therapy is crucial. The incidence of MRSA as the causative pathogen for CAP varies geographically and may be comparatively higher in some of the countries involved in the study. These considerations may explain why a large proportion of patients received ceftaroline fosamil first-line for (empirical) treatment.

As expected for the patient population, *S. pneumoniae* was the most frequently identified pathogen in this study, accounting for 24%. *S. aureus* accounted for 18% of organisms identified. This percentage is higher than is often stated in the literature, with previous studies and case series from Europe and North America typically reporting values of ∼2%–5%.^25–27^ However, considerable geographical variation in bacterial aetiology exists, with rates of 17% *S. aureus* CAP previously observed in Latin America.^28^ Additionally, in an evaluation of epidemiological data from multiple studies, the prevalence of *S. aureus* CAP was found to be 0%–1% in outpatients, 0%–4% in patients admitted to hospital, 0%–19% in those admitted to intensive care, and 7%–29% in elderly hospitalized patients.^29^ Furthermore, it has been estimated that in up to 36% of severe CAP cases no causative pathogen is identified, suggesting that the prevalence of *S. aureus* may potentially be higher than previously reported.^30,31^ Of note, *S. aureus* CAP, particularly that caused by MRSA, is associated with increased severity of disease compared with that of pneumococcal origin.^26^ Indeed, in the current study where high levels of *S. aureus* were documented, 69% of patients were categorized as having severe CAP based on prognostic scoring info and/or physician judgement, and 41% were categorized based on ICU admission.

While differences in trial design and cohorts inevitably exist between this retrospective analysis and the Phase 3 randomized, controlled clinical studies, the treatment response results observed here are nevertheless in accordance with the Phase 3 trials.^10,11,13^ In the FOCUS 1 and 2 CAP trials, ceftaroline fosamil at the standard adult dose [600 mg every 12 h by 1 h IV infusion (adjusted for patients with renal impairment)] was compared with ceftriaxone among adults hospitalized with PORT risk class III or IV CAP. Ceftaroline fosamil was non-inferior to ceftriaxone 1 g every 24 h in the individual FOCUS trials,^10,11^ and an integrated analysis of the trials demonstrated numerically higher clinical cure rates for ceftaroline fosamil versus ceftriaxone (84% versus 78%).^32^ In a further trial in Asia, standard-dose ceftaroline fosamil was superior to ceftriaxone 2 g every 24 h.^13^ Of note, patients with confirmed MRSA infection were excluded from these trials due to the inactivity of ceftriaxone against MRSA. However, findings from a recent systematic review and qualitative analysis of real-world outcomes studies suggest ceftaroline fosamil may be a possible alternative to linezolid and vancomycin for treatment of MRSA pneumonia.^33^ Ceftaroline fosamil, in combination with a macrolide or respiratory fluroquinolone, is included as a recommended empirical treatment option for non-severe and severe inpatient CAP in the IDSA/ATS CAP guidance (ERS/ESICM/ESCMID/ALAT guidance advocates for macrolides in favour of fluoroquinolones),^22^ and is also included as an option for MRSA coverage in the IDSA/ATS hospital-acquired pneumonia/ventilator-associated pneumonia recommendations.^20,34^

For any new antibiotic, it is important to assess real-world effectiveness, to allow evaluation of its use across a broad range of patients. The Clinical Assessment Program and Teflaro^®^ Utilization Registry (CAPTURE) has reported several analyses on real-world use of ceftaroline fosamil in the USA. The overall clinical cure rate in patients with community-acquired bacterial pneumonia in CAPTURE (*n* = 398) was 79%,^12^ in line with that observed in patients in the current analysis as well as in the FOCUS and ASIA CAP trials.^10,11,13^ Clinical cure rates were similar regardless of whether ceftaroline fosamil was given as monotherapy or combination therapy, or as first-line or second-line therapy. These findings suggest a potential role for ceftaroline fosamil in the treatment of patient populations who were excluded from the Phase 3 trials, including those with MRSA CAP.^12^

The observations from the present CAP study support findings from the ceftaroline fosamil Phase 3 clinical trials,^10,11,13^ as well as those gathered from real-world data,^12^ showing ceftaroline to be effective in hospitalized patients with CAP, including those with severe illness and those with bacteraemic infection. Bacteraemia increases with CAP severity and has been associated with higher mortality, although it is considered to remain underestimated in clinical practice.^35–37^ Interestingly, in an integrated analysis of the FOCUS 1 and 2 studies, the clinical cure rate was numerically higher in bacteraemic patients in the ceftaroline arm compared with the ceftriaxone arm (71.4% and 58.8%, respectively).^38^ Additionally, in a cohort of patients with *S. aureus* bacteraemia-associated CAP collected from CAPTURE, ceftaroline fosamil treatment had a high (67%) overall success rate, supporting its use as a potential treatment option for this patient population.^39^

The overall 30 day readmission rate in the current study was 11%. This is similar to results from a US retrospective chart review study of ceftaroline fosamil in patients with MRSA pneumonia, where the 30 day readmission rate was 9%.^40^ Risk factors for 30 day readmission include age, hospitalization frequency during the prior 3 months, presence of comorbidities, and home healthcare availability.^41^

First-line ceftaroline fosamil therapy was associated with numerically lower mortality rates than later lines of use. A retrospective cohort analysis of 515 patients with CAP-related bacteraemia from the US Premier database found in-hospital mortality to be numerically lower (11%) in patients receiving ceftaroline fosamil as first-line therapy versus those receiving it as second-line therapy (15%).^42^ However, real-world studies have shown ceftaroline fosamil to be effective for treatment of CAP, regardless of whether it is used as first- or second-line therapy.^12,43^ In the present analysis, 30 day all-cause mortality rates were 5/57 (9%) in patients with non-severe CAP and 13/128 (10%) in those with severe CAP. The relatively small difference in mortality rates is somewhat unexpected and may reflect the comparatively small non-severe group and/or the observational nature of the study. Moreover, as prognostic data were not available for all patients, it is possible that some might not have been assigned to the appropriate severity group. In the sensitivity analysis, using ICU admission as an objective marker, 30 day mortality rate was slightly higher at 13%.

Mortality rates in this study were overall higher than those in the FOCUS 1 and 2 clinical studies (2% and 3%, respectively),^10,11^ perhaps reflecting differences in baseline demographic and microbiological characteristics, as well as the high proportion of patients with severe disease in this study.

Clinical response to ceftaroline fosamil for treatment of CAP was associated with reductions in healthcare resource use, including shorter lengths of both hospital and ICU stay, compared with non-responders. Healthcare costs were also reduced in responders versus non-responders. Of note, similar reductions in costs were also observed in the cSSTI patient dataset (data on file; to be reported separately).

Data from CAPTURE showed that those who received ceftaroline fosamil as first-line therapy tended to have shorter lengths of hospital stays and lower associated total hospital costs.^44,45^ Furthermore, data from a cost-consequences model predicted that, in patients with CAP who responded to treatment, more would be discharged early with ceftaroline fosamil than with ceftriaxone (30.6% versus 26.1%).^46^ Of note, in the subgroup of patients with pneumococcal pneumonia, ceftaroline fosamil was cost-saving versus ceftriaxone by 1.2%, while significantly increasing the number of patients achieving initial antibiotic treatment success and early discharge (32.1% versus 24.6%).

Additionally, data from a 3 year hospital budget impact model showed a total cost saving of $1102 when treating a patient with CAP with ceftaroline fosamil versus ceftriaxone ($18 925 versus $20 027; sensitivity analysis range: −$6 to −$2223).^47^ Combined, these data support the proposal that ceftaroline fosamil may be a cost-effective treatment option in patients with CAP.

A strength of the current study is that the data obtained may be more representative of real-world use of ceftaroline fosamil in patients, compared with patients enrolled in clinical trials with restrictive study eligibility criteria. However, the retrospective design also represents a limitation of this study. Data were collected from the patients’ hospital records, with additional information unable to be collected; therefore, if not captured, data were recorded as missing for the purposes of analysis. In addition to information bias, retrospective studies may be associated with selection bias. In this study, the requirement of patients to have certain characteristics to qualify for study inclusion may have resulted in potential selection bias. Numbers of patients screened before exclusion were not available from the individual study sites, which may also have implications for selection bias.

Another potential limitation is that the requirement for patients to have had at least four consecutive IV doses of ceftaroline fosamil would have excluded some patients from the study. This requirement was included in the study protocol for alignment with the CAPTURE study. Additionally, prognostic scoring system data were unavailable for many patients and, while the patient CRF did include a section for ‘criteria for severe CAP’, a more detailed breakdown of severity criteria was not included, resulting in severity of CAP being attributed to physician judgment in these cases. Uncertainty of CAP severity in these patients thus represents a limitation of this analysis. However, sensitivity analyses conducted using ICU admission as an objective marker of severe CAP serve to at least partially mitigate this limitation. Finally, as healthcare costs differ between countries and healthcare systems, there are inherent limitations surrounding the aggregation of country-specific cost estimates. Nevertheless, the observations from this study provide information regarding economic impact of ceftaroline fosamil in a real-world setting across different geographical regions.

In summary, the results from this study provide real-world evidence of the effectiveness of ceftaroline fosamil in patients with CAP in usual care settings in Europe and Latin America. Clinical response rates were similar for both overall and severe CAP (albeit CAP severity being defined imperfectly) in this hospitalized patient population of whom most received empirical first-line ceftaroline fosamil. Clinical response to ceftaroline fosamil was also associated with shorter lengths of hospital and ICU stay compared with non-responders. These real-world data support previously reported clinical and real-world evaluations providing evidence of the feasibility of ceftaroline fosamil as an alternative treatment option to potentially improve empirical treatment success rates against a range of suspected causative CAP pathogens.

## Supplementary Material

dlae078_Supplementary_Data

## Data Availability

Upon request, and subject to certain criteria, conditions and exceptions (see https://www.pfizer.com/science/clinical-trials/trial-data-and-results for more information), Pfizer will provide access to individual de-identified participant data from Pfizer-sponsored global interventional clinical studies conducted for medicines, vaccines and medical devices (i) for indications that have been approved in the USA and/or EU, or (ii) in programmes that have been terminated (i.e. development for all indications has been discontinued). Pfizer will also consider requests for the protocol, data dictionary and statistical analysis plan. Data may be requested from Pfizer trials 24 months after study completion. The de-identified participant data will be made available to researchers whose proposals meet the research criteria and other conditions, and for which an exception does not apply, via a secure portal. To gain access, data requestors must enter into a data access agreement with Pfizer.

## Transparency declarations

W.A. and M.K. are employees of and shareholders in Pfizer. B.S. is an employee of ICON and G.L. is a former employee of ICON, who were paid consultants to Pfizer in connection with the development of manuscript. T.F., A.S., C.G., R.d.P., F.B. and M.B. received institutional research grant funding from Pfizer for the conduct of the study. F.B. reports grants and/or personal fees from AstraZeneca, Bayer, Chiesi, GSK, Grifols, Guidotti, Insmed, Menarini, Novartis, Pfizer, Zambon and Vertex outside the submitted work.

## Supplementary data

Tables S1 to S5 are available as Supplementary data at *JAC-AMR* Online.
