# Supplementary material for: Ceftaroline fosamil treatment patterns and outcomes in adults with community-acquired pneumonia: a real-world multinational, retrospective study
Source: JAC Antimicrob Resist. 2024 May 27;6(3):dlae078. doi: 10.1093/jacamr/dlae078 (PMC11128847; doi:10.1093/jacamr/dlae078)
Supplement: dlae078_Supplementary_Data [file dlae078_supplementary_data.docx]

Supplementary Material

Ceftaroline fosamil treatment patterns and outcomes in adults with community-acquired pneumonia: a real-world multinational, retrospective study

Alex Soriano^1^, Matteo Bassetti^2^, Charalambos Gogos^3^, Tristan Ferry^4^, Raul de Pablo^5^, Wajeeha Ansari^6^, Michal Kantecki^7^, Bernd Schweikert^8^, Gustavo Luna^9^ and Francesco Blasi^10,11^

^1^Hospital Clínic de Barcelona, Barcelona, CIBERINF, CIBER in Infectious Diseases, Spain; ^2^Clinica Malattie Infettive, Ospedale Policlinico IRCCS San Martino and University of Genoa, Genoa, Italy; ^3^University of Patras, Patras, Greece; ^4^Croix-Rousse Hospital, Hospices Civils de Lyon, Lyon, France; ^5^Hospital Universitario Ramón y Cajal, IRYCIS, University of Alcalá, Madrid, Spain; ^6^Pfizer, New York, NY, USA; ^7^Pfizer, Paris, France; ^8^ICON plc, Munich, Germany; ^9^ICON plc, Stockholm, Sweden; ^10^Respiratory Unit and Cystic Fibrosis Center, Fondazione IRCCS Cà Granda Ospedale Maggiore Policlinico Milano, Italy; ^11^Department of Pathophysiology and Transplantation, Università degli Studi di Milano, Italy

# Supplementary methods

# Ethics committee approval of the study protocol

| **Country** | **IEC/IRB** | **Approval date** | **Approval/ reference number** | **ICF waiver granted or ICF obtained** |
| --- | --- | --- | --- | --- |
| Brazil | Fundação Altino Ventura | 30 July 2020 | Opinion Number: 4,183,061 | ICF |
|  | Real Hospital Português de Beneficência de Pernambuco | 30 March 2021 | Opinion Number:  4,622,450 | ICF |
|  | Comissão Nacional de Ética em Pesquisa (CONEP) | 3 November 2020 | Opinion Number: 4,376,031 | ICF |
| Colombia | Ethics Committee Fundación Valle del Lilli | 22 April 2020 | 1559 (Registrado en este comite con el Numero) | Waiver |
|  | Ethics Committee Centro Médico Imbanaco | 25 March 2020 | Ref.: CEI – 468 | Waiver |
|  | Ethics Committee Universidad Tecnológica de Pereira | 13 April 2020 | GUARANTEE CODE: 01-300320 | Waiver |
|  | Ethics Committee Clínica Las Américas | 4 March 2020 | EC Minutes No. 154 | Waiver |
| France* | N/A | N/A | N/A | N/A |
| Greece | Scientific Council ATTIKON University General Hospital | 15 July 2020 | Reference No. 26074/06-08-2020 | ICF |
|  | Scientific Council University General Hospital of Patras | 27 August 2020 | Reference No.: 417 | ICF |
|  | Scientific Council AHEPA University General Hospital of Thessaloniki | 7 July 2020 | Reference No.: [HW: 459] | ICF |
|  | Scientific Council University General Hospital of Heraklion | 2 September 2020 | Reference No.: 9788 | ICF |
|  | Scientific Council University General Hospital of Larisa | 16 July 2020 | Reference no.: 26856 | ICF |
| Italy | Single Regional Ethics Committee (CEUR) | 23 September 2020 | Reference no. 0030586 / P / GEN/ ARCS | Waiver |
|  | Ethics Committee of the Campania “Luigi Vanvitelli” University | 6 March 2020 | Record no. 179 | Waiver |
|  | Ethics Committee of Brescia | 29 June 2020 | NP 3842 | ICF |
|  | Interhospital Ethics Committee of Città della Salute e della Scienza University Hospital, Turin -Mauriziano Order Hospital - Città di Torino LHA | 11 September 2020 | Reference file A/2.4.8  File no. 139/2019 | ICF |
| Russia | Hospital Local Ethics Committee of State Budgetary Healthcare Institution G. G. Kuvatov Republican Clinical Hospital | 18 March 2020 | EC Meeting Minutes No. 3 | Waiver |
|  | Biomedical Ethics Committee at the Budgetary Healthcare Institution of the Udmurt Republic “First Republican Clinical Hospital of the Ministry of Health of the Udmurt Republic” | 26 March 2020 | EC Meeting Minutes No. 2 dated 26 March 2020 | Waiver |
| Spain** | Ethics Committee Hospital Clínic de Barcelona | 29 October 2019 | Reg. HCB/2019/0962 | Waiver |
|  | N/A | N/A | NA | N/A |
|  | N/A | N/A | NA | N/A |
|  | N/A | N/A | NA | N/A |

*** Submission to IEC/IRB in France was not applicable as per the methodology MR-004 issued by the French Data Protection Authority (CNIL)

**** Remaining sites in Spain followed the approval by the lead site’s ethics committee (Hospital Clínic de Barcelona)

ICF, informed consent form; IEC, independent ethics committee; IRB, institutional review board; N/A, not applicable.

## Diagnostic criteria for CAP

1. Imaging findings of the chest consistent with a diagnosis of bacterial pneumonia at the date of admission to the hospital.

2. Acute illness at the date of admission to the hospital with at least three of the following:

- New or increased cough severity
- Purulent sputum or change in sputum character
- Auscultatory findings consistent with pneumonia
- Dyspnoea, tachypnoea or hypoxaemia (O_2_ saturation <90% on room air or pO_2_ <60 mmHg)
- Fever (>38°C oral or 38.5°C rectally or tympanically) or hypothermia (<35°C)
- White blood cell (WBC) count >10,000 cells/mm^3^ or < 4500 cells/mm^3^
- >15% bands irrespective of WBC count.

## Questions pertaining to determination of CAP severity on patient CRF

Criteria for severe CAP?

- Yes
- No
- Unknown

Which prognostic scoring system was used to evaluate illness severity?

- Patient outcomes research team risk/Pneumonia Severity Index Score: [[*free text*]] (range 0-400)
- Confusion, urea nitrogen, respiratory rate, blood pressure, and ≥65 years of age score: [[*free text*]] (range 0–5)
- Other
- None
- Unknown

*(Scores will only appear if the corresponding option is selected)*

## Definitions of sepsis and septic shock

**Sepsis**

Defined as having a known infection plus ≥2 of the following:

- Temperature ≥38◦C or ≤36^◦^C
- Heart rate ≥ 90 beats per min
- Respiratory rate ≥ 20 breathes per min or PaCO_2_ ≤32 mmHg
- White blood count ≥12,000 or ≤4,000

**Severe sepsis**

Defined as sepsis plus ≥1 the following signs of organ hypoperfusion or organ dysfunction

- Sepsis-induced hypotension
- Lactate greater than the upper limits of normal lab results
- Urine output <0.5 mL/kg hour for > 2 h, despite adequate fluid resuscitation
- Acute lung injury with PaO_2_/FiO_2_ <200 in the presence of pneumonia as infection
- source
- Creatinine >2.0 mg/dL (176.8 μmol/L)
- Bilirubin >2mg/dL (34.2 μmol/L)
- Platelet count <100,000
- Coagulopathy (INR >1.5)

**Septic shock**

Defined as presence of severe sepsis + 1 of the following:

- Systemic blood pressure <60 mmHg (<80 mmHg if previous hypertension) after

20-30 mL.kg starch or 40-60 mL/kg serum saline solution

- Pulmonary capillary wedge pressure (PCWP) between 12 and 20 mmHg and

need for dopamine of >5 mcg/kg/min

- Norepinephrine or epinephrine of <0.25 mcg/kg/min to maintain mean blood

pressure at <60 mmHg (80 mmHG if previous hypertension)

- Lactate acidosis (lactic acid>2mm/L)

## Supplementary Results

Table S1. Pre-ceftaroline fosamil treatment summary across all treatment lines

| Variable | Patients  (*n* = 185) |
| --- | --- |
| Antibiotic treatment for the index infection received prior to ceftaroline fosamil, *n* (%) | 134 (72.4) |
| Treatment received, *n* (%)^†^ |  |
| Aminoglycoside | 5 (3.7) |
| Beta-lactam | 17 (12.7) |
| Carbapenem | 12 (9.0) |
| Ceftriaxone | 45 (33.6) |
| Cephalosporin | 19 (14.2) |
| Glycopeptide | 4 (3.0) |
| Macrolide | 13 (9.7) |
| Beta-lactam/combination | 36 (26.9) |
| Quinolone | 50 (37.3) |
| Sulfonamide | 37 (27.6) |
| Clindamycin | 1 (0.7) |
| Other | 15 (11.2) |
| Number of lines of pre-ceftaroline fosamil therapy, median (range) | 2 (1–8) |
| Duration of pre-ceftaroline fosamil therapy, days, median (range) | 6 (0–45) |

^†^*n* = 117 (data not available for 17 patients).

Table S2. Ceftaroline fosamil administered as combination therapy

| Treatment variable | Patients  (*n* = 185) |
| --- | --- |
| Ceftaroline fosamil given as monotherapy/combination therapy, *n* (%) |  |
| Monotherapy | 56 (30.3) |
| Combination therapy | 129 (69.7) |
| Combination treatment received, *n* (%)^†^ |  |
| Aminoglycoside | 3 (2.3) |
| Beta-lactam | 8 (6.2) |
| Carbapenem | 7 (5.4) |
| Ceftriaxone | 5 (3.9) |
| Cephalosporin | 1 (0.8) |
| Glycopeptide | 5 (3.9) |
| Macrolide | 2 (1.6) |
| Beta-lactam/combination | 36 (27.9) |
| Quinolone | 6 (4.7) |
| Sulfonamide | 57 (44.2) |
| Clindamycin | 5 (3.9) |
| Other | 5 (3.9) |
| Doses administered, mean (SD) | 11.6 (11.7) |
| Duration of combination therapy, days, mean (SD) | 8.1 (4.8) |

^†^*n* = 99 (data not available for 30 patients).

Table S3. Post-ceftaroline fosamil treatment summary across all treatment lines

| Treatment variable | Patients  (*n* = 185) |
| --- | --- |
| Antibiotic treatment for the index infection received after ceftaroline fosamil, *n* (%)^†^ | 99 (53.5) |
| Treatment received, *n* (%)^‡^ |  |
| Aminoglycoside | 9 (9.1) |
| Beta-lactam | 13 (13.1) |
| Carbapenem | 8 (8.1) |
| Ceftriaxone | 18 (18.2) |
| Glycopeptide | 7 (7.1) |
| Macrolide | 12 (12.1) |
| Beta-lactam/combination | 27 (27.3) |
| Quinolone | 45 (45.5) |
| Sulfonamide | 12 (12.1) |
| Clindamycin | 32 (32.3) |
| Other | 12 (9.1) |
| Duration from ceftaroline fosamil discontinuation to initiation of new treatment, days, median (range) | 1 (0–56) |
| Reason for treatment modification, n^§,ǁ^ |  |
| Lack of efficacy of previous treatment | 39 |
| Side effect of previous treatment | 1 |
| Results of susceptibility test/pathogen identification | 26 |
| Other | 124 |

^†^*n* = 183 (data not available for two patients).

^‡^*n* = 90 (data not available for nine patients).

^§^*n* = 99 (multiple reasons given for some patients).

^ǁ^More than one reason for treatment modification could be provided.

Table S4. Survival status by microbial aetiology

| Pathogen (n/N [%]) | Deceased (*n* = 31) | Alive (*n* = 126) |
| --- | --- | --- |
|  |  |  |
| *Streptococcus pneumoniae* | 8/41 (19.5) | 33/41 (80.5) |
| *Escherichia coli* | 0/2 (0) | 2/2 (100) |
| *Haemophilus influenzae* | 0/3 (0) | 3/3 (100) |
| *Haemophilus parainfluenzae* | 1/1 (100) | 0/1 (0) |
| *Legionella* spp. | 0/2 (0) | 2/2 (100) |
| *Staphylococcus aureus* (methicillin susceptibility unspecified) | 1/3 (33.3) | 2/3 (66.7) |
| MRSA | 5/14 (35.7) | 9/14 (64.3) |
| MSSA | 2/12 (16.7) | 10/12 (83.3) |
| *Klebsiella pneumoniae*^†^ | 1/1 (100) | 0/1 (0) |
| Other enterobacteria | 1/2 (50.0) | 1/2 (50.0) |
| Other microorganism | 5/21 (23.8) | 16/21 (76.2) |
| Unknown | 1/12 (8.3) | 11/12 (91.7) |
| None of the above | 9/60 (15.0) | 51/60 (85.0) |

^†^Not susceptible to ceftaroline fosamil.

**Table S5.** Healthcare costs by country (local currency)

| Hospital costs (local currency), mean (SD) | Clinical response to ceftaroline fosamil^†^ | |
| --- | --- | --- |
|  | Response (*n* = 151) | No response (*n* = 34) |
| **France (EUR)** |  |  |
| Standard hospital‡ | 10,129.32 (3,993.9) | 10,774.39 (12,124.15) |
| Advanced-level hospital^§^ | 3,9425 (15,544.91) | 4,1935.71 (4,7189.22) |
| **Greece (EUR)** |  |  |
| Standard hospital‡ | 1,591.49 (680.1) | 2,466.67 (1,256.45) |
| Advanced-level hospital^§^ | 5,470.74 (2337.84) | 8,479.17 (4,319.05) |
| **Italy (EUR)** |  |  |
| Standard hospital‡ | 9,760.76 (6,699.9) | 7,592.57 (6,648.55) |
| Advanced-level hospital^§^ | 43,686.53 (29,986.95) | 33,982.29 (29,757.08) |
| **Spain (EUR)** |  |  |
| Standard hospital‡ | 16,453.18 (14,794.8) | 21,626.03 (12,440.41) |
| Advanced-level hospital^§^ | 33,594.61 (30,208.47) | 44,156.68 (25,401.21) |
| **Russia (RUB)** |  |  |
| Standard hospital‡ | 18,443.48 (6,880.00) | 14,700 (989.95) |
| Advanced-level hospital^§^ | 111,978.3 (41,771.42) | 89,250 (6,010.41) |
| **Brazil (BRL)** |  |  |
| Standard hospital‡ | 9,219.6 (4,135.31) | - |
| Advanced-level hospital^§^ | 28,130.31 (1,2617.42) | - |
| **Colombia (COP)** |  |  |
| Standard hospital‡ | 13,230.700 (10,612,366) | 6,615,350 (5,704,584) |
| Advanced-level hospital^§^ | 59,634,500 (47,832,930) | 29,817,250 (25,712,170) |

Hospital length of stay was translated into monetary terms based on country-specific unit cost, expressed in local currency. Costs were converted from local currency into USD using the exchange rate as of Aug 17th, 2021.

^†^Clinical response defined as demonstrating clinical stability (defined according to the IDSA guidelines ^20^ as temperature of ≤37.8 C°, heart rate of ≤100 beats/min, respiratory rate of ≤24 breaths/min, systolic blood pressure of ≥90 mmHg, oxygen saturation of ≥90%, and confusion/disorientation recorded as absent) and clinical improvement (defined as improvement of at least one of four symptoms present at baseline [*i.e.* cough, dyspnoea, pleuritic chest pain, or sputum production] with worsening of none).

^‡^Standard hospital cost: total time in hospital multiplied by per diem rate of standard hospital general ward.

^§^Advanced hospital cost: total time in hospital multiplied by per diem rate of hospitals providing the highest level of medical services.

BRL, Brazilian Real; COP, Colombian Pesos; EUR, Euros; RUB, Russian Rubel; SD, standard deviation; USD, US dollars
